# *Announcement: *Federally Assisted Housing Standards for Blood Lead Levels Aligned with CDC-Recommended Threshold

**DOI:** 10.15585/mmwr.mm6605a4

**Published:** 2017-02-10

**Affiliations:** 1 Office of Policy, Planning, and Partnerships, National Center for Environmental Health, CDC.

On January 13, 2017, the U.S. Department of Housing and Urban Development (HUD) lowered the threshold of lead in young children’s blood that triggers interventions to evaluate and control exposure hazards from 20 *μ*g/dL to 5 *μ*g/dL, matching the reference level used by CDC (*1*). The rule includes a process to continue HUD alignment with any future updates to CDC’s reference level (*1*).

There is no known safe level of childhood lead exposure (*2*). Lead exposure can affect nearly every body system (*2*). Even low blood lead levels can damage a child’s brain and nervous system, slow growth and development, cause hearing and speech problems, and affect intelligence quotient (IQ), academic achievement, and behavior (*3*). Lead poisoning also places a social and economic burden on families, communities, and the nation, estimated at $192–270 billion over the lifetime of the cohort of U.S. children aged ≤6 years (*3*). Lead control programs are highly cost effective: for every dollar spent, $17–$221 is returned in health benefits, increased IQ, higher lifetime earnings and tax revenue, reduced spending on special education, and reduced criminal activity (*3*).

Despite significant reductions in lead poisoning over the last several decades, homes remain the primary sources of childhood lead exposure. Approximately 24 million U.S. homes contain deteriorated lead-based paint and lead-contaminated house dust (*4*); even conservative estimates suggest that >535,000 children aged <5 years have blood lead levels high enough to damage their health (*5*). HUD estimates that 57,000 housing units affected by the rule have lead-based paint hazards and are occupied by children aged <6 years (*6*).

Additional information about childhood lead poisoning prevention is available at https://www.cdc.gov/nceh/lead/.
